# Brazos Valley Medical Association

**Published:** 1903-06

**Authors:** W. B. Briggs

**Affiliations:** Secretary; Cameron, Texas


### Society Notes.

### Brazos Valley Medical Association.

CAMERON, TEXAS, June 5, 1903.

The Brazos Valley Medical Association of Texas held their fifteenth semi-annual meeting at Cameron on May 12 and 13, 1903.

The following papers were read and discussed:

"The Acute Form of Tonsilitis," Dr. A. R. Nowlin, Cameron.

"Constipation as a Factor in Diseases of the Rectum and Sigmoid Fluxure of the Colon," Dr. J. E. Morris, Madisonville.

"Follicular Tonsilitis," Dr. W. N. Brooks, Franklin.

"Painless Treatment of the Drug and Alcohol Habit," Dr. J. Walter Torbett, Marlin.

"Therapeutic Value of Animal Extracts," Dr. A. S. Epperson, Cameron.

"Diluting and Washing the Blood in Some Cases of Acute Poisoning," Dr. W. W. Greer, Cameron.

"Some Work on the Head," Dr. R. E. B. Bledsoe, Somerville.

"Herpes Zoster and the Use of the Faradic Current," Dr. E. N. Shaw, Cameron.

"Antistreptococcic Serum in Tuberculosis," Dr. H. N. Graves, Georgetown.

Election of officers resulted as follows:

Dr. W. W. Greer, President, Cameron; Dr. J. W. Torbett, First Vice-President, Marlin; Dr. J. D. Porter, Second Vice-President, Hutto; Dr. J. W. Hudson, Treasurer, Milano; Dr. W. B. Briggs, Secretary, Easterly.

Hearne was selected as the next place for meeting. Second Tuesday and Wednesday in November.

A glorious musical entertainment, a delightful reception and a grand banquet closed the scene.

W. B. BRIGGS, Secretary.

